# The Remarkable Journey of a Weed: Biology and Management of Annual Ryegrass (*Lolium rigidum*) in Conservation Cropping Systems of Australia

**DOI:** 10.3390/plants10081505

**Published:** 2021-07-22

**Authors:** Ali Ahsan Bajwa, Sajid Latif, Catherine Borger, Nadeem Iqbal, Md Asaduzzaman, Hanwen Wu, Michael Walsh

**Affiliations:** 1 Weed Research Unit, New South Wales Department of Primary Industries, Wagga Wagga, NSW 2650, Australia; md.asaduzzaman@dpi.nsw.gov.au (M.A.); hanwen.wu@dpi.nsw.gov.au (H.W.); 2 Southern Farming Systems, New South Wales Department of Primary Industries, Wagga Wagga, NSW 2650, Australia; sajid.latif@dpi.nsw.gov.au; 3 Department of Primary Industries and Regional Development, Northam, WA 6401, Australia; Catherine.Borger@dpird.wa.gov.au; 4 School of Agriculture and Food Sciences, The University of Queensland, Gatton, QLD 4343, Australia; nadeem.iqbal@uqconnect.edu.au; 5 Weed Research Unit, The University of Sydney, Camden, NSW 2570, Australia; m.j.walsh@sydney.edu.au

**Keywords:** grain production, herbicide resistance, rigid ryegrass, weed biology, integrated weed management

## Abstract

Annual ryegrass (*Lolium rigidum* Gaud.), traditionally utilised as a pasture species, has become the most problematic and difficult-to-control weed across grain production regions in Australia. Annual ryegrass has been favoured by the adoption of conservation tillage systems due to its genetic diversity, prolific seed production, widespread dispersal, flexible germination requirements and competitive growth habit. The widespread evolution of herbicide resistance in annual ryegrass has made its management within these systems extremely difficult. The negative impacts of this weed on grain production systems result in annual revenue losses exceeding $93 million (AUD) for Australian grain growers. No single method of management provides effective and enduring control hence the need of integrated weed management programs is widely accepted and practiced in Australian cropping. Although annual ryegrass is an extensively researched weed, a comprehensive review of the biology and management of this weed in conservation cropping systems has not been conducted. This review presents an up-to-date account of knowledge on the biology, ecology and management of annual ryegrass in an Australian context. This comprehensive account provides pragmatic information for further research and suitable management of annual ryegrass.

## 1. Introduction

**Figure 1:**
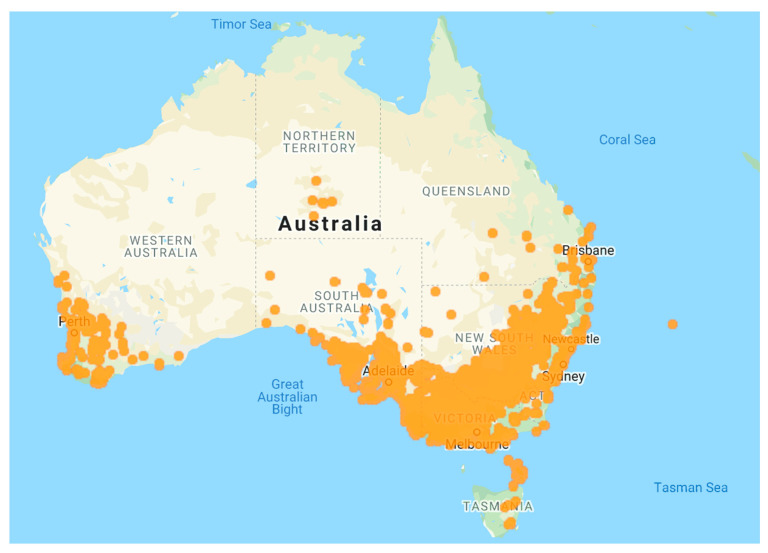
The incidence of annual ryegrass in Australia, from State herbarium records. (The data to develop the map was obtained with permission from the Atlas of Living Australia, https://doi.ala.org.au/doi/10.26197/5ea770f9b4e21 (accessed on 15 May 2021).

Australia was once one of the world’s largest wool producers. In recent decades, sheep production has markedly decreased and has been replaced with continuous grain production systems, mainly wheat (*Triticum aestivum* L.) across Australia. Unfortunately, this industry replacement of sheep with continuous grain production systems made a popular pasture species, annual ryegrass (*Lolium rigidum* Gaud.) an unwanted plant/weed [1]. Annual ryegrass is native to Mediterranean regions of southern Europe, the Indian sub-continent, northern Africa and western Asia [2]. This species had been widely cultivated across Australia in pastures and, was distributed and widely naturalised prior to the production shift to widespread grain production [3] (Figure 1). With continuous production in dairy and livestock industries, annual ryegrass continues to be planted as a pasture species, in spite of the problems it causes to grain cropping.

Australian cropping systems are among the most productive and innovative in the world [4]. Fundamental to this efficiency is the widespread adoption of conservation agriculture systems [5]. Australian growers were among the first to adopt conservation tillage; realising its resource efficiency, soil conservation potential and economic benefits [6]. The adoption of conservation tillage removed the use of cultivation and burning practices for weed control and consequently led to a reliance on herbicides [7]. A reliance on selective herbicides for in-crop weed control and non-selective herbicides for pre-seeding and fallow weed control quickly led to widespread herbicide resistance in the annual ryegrass populations that are endemic in Australia’s cropping regions.

Annual ryegrass has become the most problematic weed species in the country thanks to its widespread evolution of multiple resistance mechanisms that confer resistance to most herbicide modes of action used to control this weed. Currently, the majority (>60%) of Australian annual ryegrass populations are resistant to one or more herbicide modes of action [8]. A recent survey of Australian growers determined that this weed infests large areas of the cropping region (8 million ha) and is responsible for large yield (0.35 million tons) and revenue losses ($93 million AUD) each year [9]. Annual ryegrass was found in 81% of winter cereals, 16% of canola (*Brassica napus* L.) and pulse crops, and 3% of fallow fields. Infestations of annual ryegrass predominate in Western Australia (WA) (4.27 million ha), followed by southern Australia (3.42 million ha) [9]. In the northern cropping region, annual ryegrass has less of a presence (0.32 million ha), but it is still ranked in the top 10 weeds due to its high economic impact. About 76% of growers listed annual ryegrass as the weed most difficult and expensive to control in their cropping systems. Additionally, 83% of growers listed it as the most predominant herbicide-resistant weed [9] and this resistance is estimated to cost Australian growers an additional $103 million annually. This illustrates the enormous economic impact of annual ryegrass in Australian crop production and the ongoing need for its effective management.

Although annual ryegrass is an extensively researched weed, a comprehensive review covering major aspects of its biology, ecology and management in the context of phenomenal adoption of conservation cropping systems in Australia is lacking. This review presents an up-to-date account of the biology, ecology and management of annual ryegrass in these cropping systems. We synthesise available literature to extract and summarise key findings to advance our understanding of this weed. Information on the distribution, biology, ecology and interference the weed causes is discussed in such a way that helps establish the links between biological traits, ecological processes and management of the weed. The existing management options are discussed in terms of their relative efficacy and their future potential use to highlight future research needs.

## 2. Conservation Cropping Practiced in Australia

The practice of conservation cropping varies globally but it is often referred to the switch from inversion tillage to non-inversion tillage [10]. In Australia, the traditional method of cultivation was full disturbance using tynes to a depth of 10 to 15 cm, potentially combined with removal of crop residue [11]. Conservation cropping is defined as either minimum tillage or no-tillage practices, which buries about 90% of weed seeds to varying soil depths whereas zero tillage leaves about 95% of weed seeds on the soil surface (Table 1). Obviously, these systems vary widely, in terms of soil disturbance, weed seed burial and crop residue retention. In Australian cropping systems, many growers occasionally include full soil disturbance once every 5 to 10 years to control problematic weeds and address soil constraints (‘strategic deep tillage’) (Table 1) [12]. There is often few data available on soil movement and eventually weed seed movement due to these tillage practices and it can vary widely between soil types [12]. There are also very few data on the characteristics of crop residues in varying agronomic systems. Borger et al. [13] found 2430 to 4480 kg ha^−1^ of crop residues at Cunderdin, WA, and 1030 to 1690 kg ha^−1^ at Wongan Hills, WA, from crops with varying row spacing, harvested at varying heights, in a no-tillage system. However, these are both lower rainfall areas and the study did not compare residue from different crop species or tillage techniques. In southern Australia, over the dry summer fallow, most residue remains on the soil surface until the following cropping season [13]. Soil disturbance and crop residue will impact the environment that weeds require for establishment e.g., soil temperature, evaporation, light availability etc., and pre-emergent herbicide performance (i.e., herbicide incorporation, volatility etc.) [13,14,15]. It is clear that we need greater understanding of soil disturbance, soil characteristics and crop residue characteristics in minimum tillage, no-tillage and zero tillage systems, before we can begin to assess how weeds will respond to the altered agronomic system.

## 3. Biology and Ecology

The biology and ecology of annual ryegrass have been researched and discussed in detail, in previous reviews [1,14]. However, as detailed above, we have not fully documented the environmental changes resulting from conservation cropping adoption and their impact on annual ryegrass. Annual ryegrass is an obligate outcrossing species and pollen can travel over 3000 m between populations, ensuring high genetic diversity within and between ecotypes and rapid evolution due to agronomic changes [17]. There is little information on genetic or environmental changes in annual ryegrass induced by conservation cropping systems in Australia or if these occur at all. Studies to compare annual ryegrass in minimum tillage, no-tillage and zero tillage systems are required, to inform and allow growers to refine appropriate management strategies as necessary.

### 3.1. Seed Germination Ecology and Seedbank Dynamics

Primary seed dormancy at physiological maturity is dependent on both genetic factors and the maternal environment. Initial dormancy in WA ecotypes has been reported to range from 0 to >90% [18,19]. Plants exposed to stress in the form of warmer temperatures or reduced light availability (mainly shading by the crop canopy) produced fewer and smaller seeds with less dormancy than those from plants grown at cool temperatures or with greater light availability [20,21,22]. Plants in cool conditions with reduced growing season rainfall produced seeds that lost dormancy faster than plants in cool conditions with adequate moisture [21,22]. Seeds with primary dormancy require at least 2000 growing degree days of after-ripening, prior to germination [16,19]. The rate of dormancy loss increases with time and temperature. However, very high temperatures (>50 °C) such as experienced on the soil surface can also increase rates of seed aging or decay [1]. Below a base temperature of 5.4 °C, after-ripening does not occur. The dormancy release rate increases with the water content of the seeds from 6 to 18%, although this process can commence at water contents of close to 0% [18].

Some populations exhibit low primary dormancy, but all freshly harvested seeds have more stringent germination requirements than those seeds that have lost dormancy through after-ripening. Maximum germination of fresh seeds requires light, with approximately 32 to 85% of the initial non-dormant seed having a light requirement for germination [19]. Maximum germination of fresh seed also requires alternating temperatures, with germination inhibited below 5 °C or above 35 °C [1]. Following after-ripening, germination requirements for seeds without primary dormancy become more flexible. Maximum seed germination can occur in the dark and at a constant temperature (above the base temperature of 5.4 °C), although the optimal temperature for germination is 20 to 26 °C [18,23]. Full hydration of seeds (i.e., sufficient rain in summer to wet seeds followed by hot, dry conditions) increases the rate of dormancy release [24]. Even a partial hydration event (a small amount of rainfall) can increase seed germinability [25]. However, high rainfall at the beginning of summer (December), directly after the seeds reach maturity, can reduce the rate of dormancy loss during after-ripening over the subsequent months [22]. From a practical point of view, in southern Australia, this indicates that little or no rainfall over the summer/autumn fallow period will result in staggered germination during the winter growing season, with lower total germination. In contrast, mid to late summer rainfall events would ensure increased and more rapid germination after the first substantial rainfall event of the winter growing season.

Hydrated seeds that are buried (i.e., seeds in dark conditions) also lose primary dormancy, allowing a higher proportion to germinate when they once again experience conditions with light and variable temperatures [1,26]. However, seeds that lose dormancy during burial, unlike seeds that experience after-ripening on the surface (in warm, dry conditions), still have a light requirement for germination. There is no research on what volume of crop residue (if any) is high enough to equate to ‘burial’, but seeds under dense residue are likely in dark conditions similar to burial and these seeds may be more likely to achieve hydration as the crop residue would reduce evaporation. These seeds may be exposed to light when the residue shifts (i.e., from planting operations) and there is little research on emergence patterns under these conditions.

As discussed, conservation cropping systems in Australia carry a large volume of crop residue on the soil surface over summer [13,15]. This residue is likely to have a large impact on annual ryegrass seed after-ripening, dormancy release, seed survival and subsequent emergence patterns. For example, residue will reduce soil temperatures, potentially increasing the time for after-ripening and dormancy release [15]. The residue will reduce light availability, potentially reducing the emergence of new seeds with low primary dormancy during summer rainfall [27]. There will also be reduced evaporation following out of season rainfall events, which may increase the likelihood of full hydration of seeds, affecting subsequent dormancy loss. The research comparing the influence of the differing levels of soil disturbance and residue levels in minimum, no- and/or zero tillage systems on seedbank distribution and persistence is limited and inconclusive. Continuous cropping systems have been selected for higher dormancy in annual ryegrass populations compared to more disruptive systems with a pasture phase or break crops [28].

Annual ryegrass has a short-lived dormant soil seedbank compared to other species, but a small proportion of seed remains dormant for future years [18]. In South Australia (SA), the dormant proportion of seeds produced after 12 months ranged from 4 to 16% [29]. In field conditions in WA, a very small proportion of the seeds (1.5%) persisted up to four years [30]. Seedbank persistence depends on burial depth. Seeds in a no-tillage system in SA, where the only soil disturbance was due to crop planting, had 48 to 60% seed mortality. By comparison, a minimum tillage system, with full cultivation, twice, to a depth of 10 cm prior to crop seeding, had 12 to 39% seed decay per season [14]. For buried seeds, germination plays a greater role in seed loss compared to seeds on the soil surface. For example, emergence in SA was 49% for seeds buried at 1 cm, compared to 16% of seeds on the soil surface [29]. As a result, emergence was greatest in minimum tillage, followed by no-tillage and zero tillage systems [14,15]. The response to burial may impact emergence patterns during the season. Annual ryegrass emergence in SA soils in a minimum tillage system reached 50% a week earlier than seeds in the no-tillage system [14]. If burial in minimum tillage results in high germination, the resulting seedlings can all be controlled with pre-seeding or selective herbicides. In a zero-tillage system, emergence may be more staggered during the cropping season, making in-crop control difficult. More research is required on seed decay and emergence patterns in varying conservation cropping systems, with reference to levels of crop residue.

### 3.2. Life Cycle and Population Dynamics

Annual ryegrass seeds with no primary dormancy may emerge in response to sporadic summer rainfall [19]. Summer cohorts are common in SA and southern WA, although these plants produce fewer seeds with reduced dormancy, compared to the winter plants [21,31]. The after-ripening requirements discussed above ensure that the bulk of emergence occurs at the first significant rainfall event of the winter growing season, often at the same time as the winter grain crop. Dry sowing is becoming increasingly common in southern Australia due to increased farm size and variable climatic conditions, i.e., dry sowing was practiced on 71% of farms with an area greater than 5000 ha of cropping [32]. There is speculation that earlier sowing combined with slower-developing wheat genotypes exploiting a longer growing season could allow increased yield in the face of climate change [33]. The base temperature for the growth of both annual ryegrass and winter grain crops in Australia is 0 °C, allowing growth under similar climatic conditions [34]. Therefore, dry sowing will make it increasingly common for annual ryegrass to emerge at the same time as the crop (i.e., which has no non-selective control prior to seeding) but emergence may be staggered or delayed in a zero tillage system [14,35].

The initially dormant seeds require a minimum of 2000 growing degree days to germinate and up to 6000 growing degree days to reach 90% germination; allowing smaller, staggered cohort emergence throughout the winter/spring growing season [19,34,36]. Cohorts emerging later in the growing season are subjected to higher ambient temperatures and increased crop competition leading to reduced seed production [37,38]. A recent study from Queensland indicated that annual ryegrass emerging at 3 or 6 weeks after planting a chickpea (*Cicer arietinum* L.) crop had a biomass of 4.7 to 22.2 g m^−2^ and 5 to 24 seed heads m^−2^, whereas plants that emerged at 0 weeks after planting had a biomass of 282 to 337 g m^−2^ and 89 to 120 seed heads m^−2^ [37]. In conservation cropping systems, crops frequently emerge following the initial significant seasonal rainfall event. As a result, there is a large proportion of seasons where annual ryegrass emergence occurs at the same time as the crop and these plants will be more competitive, with greater seed production.

Seed germinating on the soil surface will grow into a less vigorous plant with lower seed production compared to the plants resulting from seeds that germinated from a position of shallow burial [39]. In the minimum or no-tillage system, most seeds will be buried at an ideal depth to grow into vigorous plants. By comparison, in a zero-tillage system, most seeds emerge on the soil surface and the resulting plants may have reduced seed production. There is little research on initial emergence times of annual ryegrass in conservation cropping systems [14]. Also, there is no information on the plant growth rate, size and seed production in zero tillage, no-tillage or minimum tillage systems, at varying weed density and in the presence or absence of herbicides.

A survey of annual ryegrass in wheat fields (predominantly conservation cropping systems) across southern Australia indicated that plants grew at a density of 1 to 51 plants m^−2^ and produced 87 to 7192 seeds m^−2^ [40]. Most mature annual ryegrass seeds remain attached to the flower stem [41,42,43]. In WA, at the earliest opportunity for crop harvest, annual ryegrass plants retained approximately 85% of seeds and 28 days later 63% [43,44]. This weed density and seed production can be influenced by time of sowing, crop species and crop competitive ability, discussed in more detail as management techniques below [45,46,47].

There are few data on how conservation cropping has impacted the height of annual ryegrass. Within crops, plants are erect but may be prostrate in a sparse, uncompetitive crop or along the fence line [42]. In 71 fields in southern Australia, the percent of annual ryegrass seed heads (inflorescences) above 40 cm ranged from 0 to 85%, as wheat biomass increased from approximately 1500 to 2500 kg ha^−1^ [40]. However, seed heads were at different levels of the crop canopy, with 21 to 33% of seed heads less than 10 cm high. There are no data to relate plant height to crop residue in conservation agricultural systems. We know standing and prostrate crop residue can affect herbicide coverage and resulting annual ryegrass control [48]. However, it is likely that dense standing crop residue shades weeds and encourages taller growth in the same way that shade from a crop impacts weed height [49]. Weed height impacts control tactics like herbicide coverage and harvest weed seed control (HWSC) [40,48,49]. Research is needed to determine if there have been genetic or environmentally induced changes to annual ryegrass height in fields with high densities of standing or prostrate crop residue in the conservation agricultural systems.

The annual ryegrass population growth rate is highly dependent on weed control measures. In the absence of herbicide use in a no-tillage (knife points and press wheels) wheat crop at Wongan Hills, WA, from 2016 to 2018, annual ryegrass density and seed production increased from 63 plants and 9137 seeds m^−2^ to 405 plants and 42,766 seeds m^−2^ [50]. A rotation of field pea (*Pisum sativum* L.), wheat and barley (*Hordeum vulgare* L.) was conducted in SA from 2010 to 2012, using minimum tillage, with varying herbicide treatments. In the field peas in 2010, applications of trifluralin (pre-plant), trifluralin and clethodim (four-leaf) or trifluralin, clethodim and glyphosate (milk to soft dough growth stages) resulted in 98, 12 and 13 annual ryegrass plants m^−2^. Following varying herbicide treatments in the 2011 wheat, the final average annual ryegrass density in the three rotations in the 2012 barley was 319, 105 and 32 plants m^−2^ [51]. This highlights that increased application of herbicides per rotation can reduce annual ryegrass density, but the population density was still increasing under all possible management regimes. In comparison, a minimum tillage trial at Merredin, WA from 2003 to 2013 (using non-selective, pre-seeding and post-emergent herbicides in each year) had annual ryegrass seed production reduced from 324 to 2 seeds m^−2^ at 9 cm row spacing and from 382 to 171 seeds m^−2^ at 36 cm row spacing, over the 11 years [52]. Where crop residue was burnt prior to crop seeding each year, the annual ryegrass seed production was reduced to 0 seeds m^−2^ in row spacings of 9 to 36 cm. Furthermore, modelling with the Weed Seed Wizard decision support tool indicated seed production would have been reduced from 80 to 10 seeds m^−2^ with HWSC in every alternate year [36,52].

Clearly, annual ryegrass is difficult to control in some rotations in conservation cropping, but effective management is feasible. There is little research on long-term weed control strategies in no-tillage or zero tillage conservation cropping systems and it is clear that further research is needed to identify crop rotations that allow highly effective control of annual ryegrass under reduced soil disturbance.

## 4. Interference with Crop Production

### 4.1. Resource Competition

Annual ryegrass populations are extremely competitive and can severely reduce crop growth, yield and quality. Significant yield losses have been reported in several winter crops including wheat, barley, field pea and canola [3,46]. Uncontrolled annual ryegrass reduced the productivity of certain wheat cultivars by up to 80% [53]. A study by Wu, et al. [54] recorded a reduction of 50% in wheat yield with an infestation of 200 annual ryegrass plants m^−2^ and an estimated cost of $250 ha^−1^. Lemerle, et al. [55] recorded up to 50% yield loss in wheat after the early establishment of annual ryegrass. A reduction of 10 to 55% in grain yield of various cultivars of barley was reported due to annual ryegrass competition [46]. A significant reduction of crop yield has been reported in canola with delayed weed control measures [56]. However, some varieties of canola experienced less yield impact even at an annual ryegrass density of 450 plants m^−2^ [56]. Differences in competitiveness from a single species of the crop can be attributed to varietal differences, agronomic practices and environmental variation. It is important to note that there are not many studies reporting direct yield losses caused by annual ryegrass in different crops under different types of conservation tillage and planting methods. Such information is important to assess the competition dynamics in order to select appropriate management tools.

### 4.2. Allelopathic Effects

Allelopathy is an important eco-physiological phenomenon contributing towards the competitive ability of many weed species including annual ryegrass. San Emeterio, et al. [57] evaluated the effect of seed, root and shoot extracts of annual ryegrass on Italian ryegrass (*Lolium multiflorum* Lam.), cock’s-foot (*Dactylis glomerata* L.) and alfalfa (*Medicago sativa* L.) seedlings. Seedling growth was significantly suppressed, although extracts had little impact on the germination of the tested species. The seed extracts exhibited a stronger inhibitory effect than the shoot extracts of annual ryegrass. Another study revealed that root elongation of wheat seedlings was more sensitive than wheat shoot length to root extracts of annual ryegrass [58]. However, increasing weed seedling density showed a greater inhibitory effect regardless of the tissue type used in agar-based bioassays [58]. Moore, et al. [59] later employed an Equal Compartment Agar Method to study density dependent phytotoxic effects of annual ryegrass against major winter crops. They reported 6 to 57%, 4 to 37% and 5 to 84% inhibition of root length in wheat, barley and canola, respectively. The use of activated charcoal and removal of the donor plant in a similar bioassay setting confirmed the association of allelopathy with growth suppression rather than resource competition. Canals, et al. [60] studied the autotoxicity of annual ryegrass and reported significant allelopathic effects on its germination and seedling growth from aqueous extracts of shoot and root tissue. Leaf extracts posed greater inhibitory effect compared to root tissue, and germination was more sensitive compared to the seedling growth.

The bioactivity observed under controlled conditions is difficult to accurately assess under field conditions due to the complexity of rhizosphere interactions [61,62]. It is difficult to separate plant growth suppression caused by allelopathy or resource competition in the field. A comprehensive evaluation of weed suppression under both field and laboratory conditions using advanced metabolomics tools can be a way forward [63]. A better understanding of the relative contribution of annual ryegrass allelopathy and competitive ability may help in developing better management practices for this species.

### 4.3. Herbicide Resistance

Annual ryegrass is notorious for its ability to evolve resistance to herbicides. Globally, it has evolved resistance to 11 distinct herbicide modes of action groups in 12 countries [64]. Most of the first cases of resistance to different modes of action were reported from Australia (Table 2) and Australia still has the largest number of cases [64].

The resistance to acetyl coenzyme A carboxylase (ACCase) inhibitor and acetolactase synthase (ALS) inhibitor herbicides is widespread throughout conservation cropping systems in Australia mainly because these modes of action have been used for the past four decades [65,66,67,68,69,70]. There are significant differences in the incidence of herbicide resistance between states and regions, with extremely high levels of resistance to aryloxyphenoxypropionates (fops) and sulfonylureas (96 to 98%) in WA [69]. Such regional differences are often due to the varied levels of cropping intensity between regions [66,71]. Higher cropping intensities are likely to place higher selection pressure on annual ryegrass, thereby resulting in higher incidence of herbicide resistance in annual ryegrass [67].

The world’s first case of annual ryegrass resistance to glyphosate (5 enolpyruvyl shikimate-3 phosphate (EPSP) synthase inhibitor) was reported in 1996 from a cropping field in northern Victoria [72,73] followed by another one from an orchard in central New South Wales (NSW) [74]. Glyphosate resistance in annual ryegrass is low in Australia, with 5 to 7% resistance in WA (Table 3), indicating that glyphosate is still an effective option for most annual ryegrass populations [66,69,71,75]. The introduction of glyphosate-tolerant crops (cotton; *Gossypium hirsutum* L. and canola) in Australia enables the use of glyphosate both in-crop and during fallow periods, which could exacerbate the risk of developing resistance to glyphosate. The resistance to another non-selective herbicide, paraquat (Photosynthesis/Photosystem I (PS I) inhibitor) has evolved in the past decade [64]. Paraquat has been successfully used in a ‘double-knock’ strategy (glyphosate closely followed by paraquat in the same season) to control annual ryegrass (especially glyphosate resistant populations) in conservation systems of Australia [76]. A complete picture of resistance status to paraquat and some other herbicides such as prosulfocarb (lipid synthesis inhibitor) and pyroxasulfone (very long chain fatty acids (VLCFA) inhibitor) in annual ryegrass in Australia remains unknown as these herbicides have not been screened in the previous resistance surveys [65,66].

The resistance of annual ryegrass to some other modes of action is currently relatively low (Table 3) partly due to the limited use in a narrow range of rotational crops [69]. However, the number of annual ryegrass populations with complex cross and multiple resistance to different herbicides within and between herbicide groups is on the rise, further restricting the effective control options [77]. Multiple resistance to ACCase- and ALS-inhibitors is widespread across the grain growing areas of southern Australia [65,66,69]. Multiple and cross resistance to other herbicide groups (microtubule assembly inhibitors, lipid synthesis inhibitors and VLCFA inhibitors) is also emerging [78,79]. Hence, diversified control programs integrating non-chemical options are needed to slow the evolution of herbicide resistance, thereby extending the commercial life of many valuable herbicides. 

**Table 2:** Herbicide resistance cases to different modes of action in annual ryegrass first reported in Australia.

Herbicide Group	Subgroup	Herbicide	Year First Reported	References
Fat synthesis/Acetyl coenzyme A carboxylase (ACCase) inhibitors	Aryloxyphenoxypropionates	Diclofop-methyl	1982	[80]
	Cyclohexanediones	Sethoxydim	1982	[64]
Acetolactate synthase (ALS) inhibitors	Imidazolinones	Imazapic/imazpyr	1982	[64]
	Sulfonylureas	Chlorsulfuron, sulfometuron	1986	[81]
Microtubule assembly inhibitors	Dinitroanilines	Trifluralin	1982	[64,82]
	Benzamides	Propyzamide	2018	[78]
Lipid synthesis inhibitors	Thiocarbamates	Triallate/prosulfocarb	1982/2018	[64,78]
Very long chain fatty acids (VLCFA) inhibitors	Chloroacetamides	Metolachlor/metazachlor	1982/2019	[64,79]
	Isoxazoline	Pyroxasulfone	2018	[78]
5-enolpyruvylshikimate-3phosphate (EPSP) synthase inhibitors	Glycines	Glyphosate	1996, 1998	[72,74]
Group Q (Inhibitors of carotenoid biosynthesis unknown target)	Isoxazolidinones	Clomazone	1982	[64]
	Triazoles	Amitrole	1988	[83]

**Table 3:** Incidence of herbicide resistance across five major modes of action herbicide groups in Australia.

Herbicide Group	Subgroup	Resistance (%)
ACCase inhibitors	Aryloxyphenoxypropionates	81 [66], 56 [67], 96 to 98 [69], 18 [84]
	Cyclohexanediones	65 [69], 1 [84]
ALS inhibitors	Imidazolinones	65 [66], 38 [67], 7 [84]
	Sulfonylureas	70 [66], 53 [67], 96 to 98 [69], 24 [84]
Photosystem II inhibitors	Triazines	2 [69], 0 to 1 [66,71,84,85]
Microtubule assembly inhibitors	Dinitroanilines	27 to 33 [71,84], <6 [65,66,84]
EPSP synthase inhibitors	Glycines	5 to 7 [69,75], 4 [85], 0 to 1 [66,84]

## 5. Management

Management of annual ryegrass has largely been impacted by the rapid adoption of conservation cropping systems. This section provides a detailed account of the existing management options and their efficacy as affected by farming systems, in general and by conservation cropping systems, in particular.

### 5.1. Chemical Control

The lack of suitable alternative weed control options for Australian conservation cropping systems has led to a reliance on herbicides to control annual ryegrass. Since the 1970s, herbicides have been the primary tool used to control annual ryegrass due to their high efficacy, convenience and cost effectiveness. Out of the currently available 20 modes of action groups, herbicides from 12 groups have been used to control annual ryegrass.

#### 5.1.1. Pre-Sowing Non-Selective (or ‘Knockdown’) Herbicides

Glyphosate and paraquat are commonly used to control annual ryegrass before sowing [86]. After the season break, a substantial amount of annual ryegrass emerges [87] and these knockdown herbicides can achieve a high level of control prior to sowing [76,88]. These two herbicides are often rotated either within a season as a ‘double-knock’ or between seasons to delay herbicide resistance [76]. Over the last decade, “dry sowing” before the season break has gained popularity to deal with climatic variability and to maximise yield potential [32,33]. However, this approach prevents the use of pre-sowing knockdown herbicide treatments and places increased pressure on the efficacy of pre-emergent herbicides and potentially a greater reliance on in-crop selective herbicides.

#### 5.1.2. Pre-Emergent Herbicides

Loss of efficacy of the selective ACCase and ALS inhibitor herbicides has forced growers to use pre-emergent herbicides, such as trifluralin [88]. There is a broad range of pre-emergent herbicides being used for annual ryegrass control in various crops [89,90,91,92]. These herbicides, from distinct groups, are commonly used in rotation between years to manage annual ryegrass populations resistant to trifluralin and to minimise resistance evolution [78,89,92]. Clearfield^®^ crops (canola, wheat and barley) and triazine-tolerant (TT) canola allow the use of imidazolinones and Photosystem II Inhibitor herbicides to control annual ryegrass, respectively. Some new herbicides have recently been registered for pre-emergent control of annual ryegrass in Australia, including cinmethylin, with a unique mode of action (fatty acid thioesterases inhibitor) [93].

#### 5.1.3. Post-Emergent Herbicides

Post-emergent control of annual ryegrass has relied heavily on the use of ACCase- and ALS-inhibitors. However, the use of these two groups of herbicides has been declining due to the widespread resistance issues discussed above. A pre-packed mixture of prosulfocarb and s-metolachlor (VLCFA inhibitor) is now commonly used as an early post-emergent option for annual ryegrass in wheat and barley. Other post-emergent options include the use of ‘imi’ herbicides in Clearfield^®^ crops, triazine herbicides in TT canola and glyphosate in Roundup Ready^®^ crops. However, the widespread resistance of annual ryegrass to ACCase- and ALS-inhibitors in conservation cropping systems has shifted herbicide use patterns toward pre-emergent and pre-sowing knockdown herbicides.

#### 5.1.4. Pre-Harvest Crop-Topping

Applying a non-selective herbicide near to the time of crop maturity, commonly known as crop-topping, is also practiced in Australia. If used correctly, crop-topping can control weed survivors and significantly reduce seed viability and seedset in annual ryegrass [51,88,94]. Currently, paraquat, diquat and glyphosate are used as crop-topping agents to control the annual ryegrass seedset close to harvest in many pulse crops. Diquat is recommended for use in wheat, barley and canola. Glyphosate is registered in wheat, but it is not registered in malting barley and only certain glyphosate formulations are registered for use in canola [95]. It is imperative to follow label recommendations and the required withholding periods when crop-topping to avoid residue violations in harvested grains.

Overall, in Australian grain cropping systems, herbicides of distinct groups are often used in tandem and applied sequentially to target annual ryegrass from pre-sowing to seedset [51,95]. Also, there has been a trend of using herbicide mixtures and rotating herbicides from different modes of action within or between seasons to control annual ryegrass and to delay the evolution of herbicide resistance [94,96,97].

### 5.2. Cultural and Physical Control Methods

The use of different cultural, agronomic and physical methods has long been successful in controlling weed species in agro-ecosystems. Some of the pre-herbicide cultural tactics are again gaining popularity due to widespread herbicide resistance evolution in annual ryegrass in conservation systems. Therefore, it is important to revisit the potential of such practices to further evaluate their efficacy under current and future management and climatic conditions.

#### 5.2.1. Grazing

Grazing is an effective management tool to control annual ryegrass infestations in pastures or winter crops, as sheep (*Ovis aries*) can readily graze annual ryegrass at all growth stages. This species is highly palatable and unlike many other grass species, annual ryegrass seeds cannot cause injury or contaminate wool or carcasses [98]. Seeds easily lose viability when ingested by sheep [99]. Grazing pastures at a stocking rate of 15 sheep ha^−1^ reduced annual ryegrass density by around 80% in the succeeding crops and pastures in Victoria [100]. Similar trends were reported in other studies (Table 4). Late grazing during summer and autumn was found to be ineffective and resulted in only a 20% reduction in seedbank of annual ryegrass as a result of seed shedding (~70%) in the field [99]. However, grazing is not always feasible due to certain management and economic issues.

#### 5.2.2. Sowing Time Adjustment

Delayed sowing was widely used by growers before the availability of selective herbicides (when the emerged weed seedlings would be killed via cultivation or non-selective herbicides). Early season rainfall events are critical factors to the success of this approach. If there is rainfall, the subsequent high levels of annual ryegrass emergence (~80%) can be controlled prior to crop planting [87]. A reduction in annual ryegrass density of 11 to 30% was recorded with each week’s delay in sowing (Table 4). This practice has some adverse effects on crop yields due to the shortening of the growing season. An estimated yield loss of 15 to 50 kg ha^−1^ day^−1^ due to delayed sowing in wheat crops has been reported [116]. Thereby, a risk assessment of the specific crop or variety should be conducted before using this method. Early crop sowing is becoming more popular due to incremental benefits in crop yields. Furthermore, the size of farms in Australia makes it logistically impossible to sow the entire farm following rainfall; the sowing operation may take several weeks. Hence, growers are reluctant to use this technique and instead, rely on pre-emergent herbicides.

#### 5.2.3. Tillage Options

Shallow tillage before crop sowing is highly effective in reducing the density and seedbank reserves of annual ryegrass. Timely cultivation can not only break dormancy but also put weed seeds in an ideal germination environment for maximum emergence and subsequent control [117]. Different studies have reported a reduction of 37 to 63% in annual ryegrass density by using shallow tillage (i.e., ‘autumn tickle’, Table 1 and Table 3). However, sandy and dry soils are not ideal for this technique. An autumn or winter tickle prior to sowing encourages fast and uniform germination of annual ryegrass seeds but relies on an initial rainfall of at least 20 mm otherwise the tillage event could cause erosion [118]. As the intensity and frequency of rainfall patterns are extremely variable in autumn, the efficacy of this technique is highly variable. Moreover, high adoption of no-till practices and the increasing use of pre-emergent herbicides has reduced the usage of this technique.

Deep inversion tillage was found to be more effective in controlling annual ryegrass as compared to shallow tillage [119]. Inversion tillage causes more disturbance and buries the seeds of annual ryegrass at a depth from which seedlings cannot emerge [14,15]. Several studies reported a reduction of 73 to 100% in plant density and the soil seedbank of annual ryegrass (Table 4). However, conventional tillage practices are not preferred in the current, predominantly no-tillage cropping systems in Australia. The strategic use of inversion or deep tillage once every 5 to 10 years is often practiced to address other soil constraints and could be useful to bury the shallow seedbank of annual ryegrass as occurs in conservation cropping systems [120].

#### 5.2.4. Other Methods

The use of other cultural techniques such as hay and silage making, mowing, manuring and mulching has also shown promise in controlling annual ryegrass (Table 4). However, the relative efficacy of these tactics depends on several agronomic, climatic and economic factors. The use of cover crops has also been suggested as an effective way to manage annual ryegrass in Australia. For example, Flower, et al. [121] reported significant control of annual ryegrass by including black oat (*Avena strigosa* Schreb.) crop in the cereal crop rotation for two consecutive seasons. Diversified rotations allowed a greater range of herbicide options and cultural techniques to control resistant populations of annual ryegrass, while being highly profitable [122]. Similarly, diversified crop rotations have shown success in reducing annual ryegrass pressure in crop production systems. Some pasture species like alfalfa and serradella (*Ornithopus sativus* Brot. cv. Cadiz) helped control resistant biotypes of annual ryegrass when rotated with wheat, barley and lupin (*Lupinus albus* L.). Doole et al. [123] reported that sequential incorporation of alfalfa in a regular rotation was an effective and economically feasible way to manage herbicide-resistant annual ryegrass. The inclusion of break crops such as herbicide tolerant canola, lupin, or field pea in a continuous wheat rotation proved highly effective in reducing in-crop weed densities and long-term soil seedbank of annual ryegrass [124].

Innovative technologies such as microwave soil heating have been found to be effective in killing annual ryegrass seeds in the upper soil layer. Microwave heating in dry soil required more irradiance time when compared to wet soil to kill the seed. Brodie et al. [125] demonstrated that microwave heating in dry soil required 12 min exposure as compared to 4 min in wet soil for 100% seed kill. Furthermore, microwave heating in dry soil killed only those seeds which were close to the soil surface as compared to wet soil, where seeds were killed up to 5 cm depth from irradiance [125]. Recently, Brodie et al. [126] reported that post-emergent application of microwaves at 400 to 500 J cm^−2^ killed all exposed seedlings of annual ryegrass and other weeds. Similar results were reported by Khan, et al. [127], suggesting that this technology may be used in Australian production systems in the future. However, currently, the machinery is expensive to power and very slow. The use of low energy laser application has also shown promising results in controlling (up to 100% control) annual ryegrass plants at early growth stages [128]. Further research is needed to upscale and make this technology efficient and affordable. In general, with current new technology developments enabling specific weed targeting, there is a need to re-evaluate alternate weed control techniques.

### 5.3. Crop Competition

The use of enhanced crop competitive ability to manage annual ryegrass has proven to be successful. It is achieved through the selection of competitive/suppressive crops and/or cultivars, high seed rate/planting density, narrow row spacing and altered row orientation [129].

#### 5.3.1. Competitive Cultivars

Competitive crop cultivars are efficient in utilizing resources available for plant growth due to several characteristics including fast growth habit, tall stature, increased leaf area, efficient root systems, faster closing canopy architecture and superior physiological activity. Studies have demonstrated that competitive cultivars of wheat, barley and canola not only effectively suppressed annual ryegrass but also yielded higher than their less competitive counterparts [46,53,130]. For instance, competitive cultivars of wheat (cv. *Dollarbird* and cv. *Katunga*) reduced annual ryegrass biomass by 50 to 57% and increased yield by 100%, compared with less competitive cultivars (cv. *Rosella* and cv. *Shrike*) grown under the same climatic and edaphic conditions in NSW [53]. A comparative analysis of multiple genotypes of bread and durum wheat (*Triticum durum* Desf.) revealed that genotypes with greater tillering, biomass accumulation and leaf area were superior in annual ryegrass suppression compared to less vigorous genotypes [53]. Lemerle, et al. [131] reported that canola hybrids were better at suppressing growth, biomass accumulation (up to 50%) and seed production of annual ryegrass when compared with open-pollinated varieties.

Erect-growing barley cultivars were more competitive against annual ryegrass when compared with dwarf cultivars with prostrate growth in WA [132]. Similarly, better suppression of annual ryegrass growth and biomass accumulation by taller cultivars of field pea have been reported from NSW and SA [133,134]. Recently, Mwendwa, et al. [135] reported significant genotypic variations in terms of the suppression of annual ryegrass and other weed species in different winter cereals and canola. The competitive cultivars of different crops outcompeted annual ryegrass by efficient nutrient and water acquisition resulting in high biomass accumulation at a faster growth rate. Breeding programs for Australian grain crops are attempting to integrate such traits to create cultivars that are not only high yielding but are also highly suppressive against problematic weed species such as annual ryegrass [136,137].

#### 5.3.2. High Seed Rate/Crop Density

The use of high seed rates to achieve higher crop densities per unit area to enhance crop competition ability has been successful against annual ryegrass. Studies from NSW and WA have demonstrated the potential of high wheat crop densities in suppressing annual ryegrass growth and seed production [55,132,138,139,140,141]. For instance, increasing wheat density from the recommended 100 to 200 plants m^−2^ halved the annual ryegrass biomass (m^−2^) and increased grain yield by 6% [138]. Dear, et al. [142] evaluated the competitive ability of five forage legume species against annual ryegrass under controlled conditions. The small-seeded legumes such as Balansa clover (*Trifolium michelianum* Savi.) and berseem clover (*Trifolium alexandrinum* L.), sown at high seed rates, were the most effective in suppressing annual ryegrass biomass due to faster growth and rapid canopy closure [142]. Increasing the density of lupin has also proved beneficial in reducing annual ryegrass growth and biomass [143].

#### 5.3.3. Narrow Row Spacing

There is clear evidence of successful suppression of annual ryegrass by narrowing the spacing between rows in grain crops in Australia. For instance, the suppression of annual ryegrass was greater in a wheat crop sown at 18 cm row spacing compared to a crop sown at 36 cm row spacing with the same seed rate used in both cases [144]. The same row spacings in field pea had a similar effect on annual ryegrass density and growth [133]. In a recent study, Mahajan et al. [37] evaluated the effect of row spacing and cultivar on annual ryegrass competition in chickpea. It was reported that narrow row spacing (25 cm) provided a 16 to 52% reduction in biomass and 26 to 48% reduction in the number of spikes of annual ryegrass as compared with wide row spacing (75 cm) depending upon the time of weed emergence [37]. The greater weed suppression at narrow row spacing also resulted in a 20% grain yield increment [37]. An 11-year trial at Merredin, WA, demonstrated the long term value of narrow row spacing of the wheat crop [52]. All crops in the rotation were sown at 9, 18, 27 or 36 cm and the annual ryegrass seed production at these row spacings was 58, 78, 223 and 333 seeds m^−2^, respectively averaged over the entire period. Furthermore, average crop yield increased from 1492 to 1658 kg ha^−1^ as row spacing reduced from 36 to 9 cm.

Narrow row spacing allows the early crop canopy closure and, therefore, reduces the light interception for weeds growing between the rows [52,145]. Furthermore, increased yield resulting from narrow row spacing, even in the absence of weeds, is well established in Australia [145]. However, narrow row spacing may also lead to increased pest and disease infestations in some crops. There are logistical issues with the management of higher crop residue when seeding the subsequent crop, if using narrow row spacing in high yielding areas [52,145]. Therefore, further research is required to optimize the use of this important cultural weed management tool.

#### 5.3.4. East–West Row Orientation

Interestingly, cropping geometry has an influence not only on crop growth and development but also on weed competition. For example, changing the orientation/direction of crop rows at the time of sowing can significantly alter the light interception dynamics which affect the weed growth and development [129]. The research from WA over the past several years has demonstrated that changing the orientation of various winter grain crop rows from conventional north–south to east–west (at a right angle to sunlight) suppressed weeds. The underlying concept is that crop plants growing in east–west rows intercept light while causing greater shading between the rows, which reduces the growth of weeds growing in that space. Borger, et al. [146] reported that east–west rows reduced the biomass of weeds including annual ryegrass by up to 51% as compared with north–south rows in wheat and barley crops. It was thought that the crop canopy in east–west rows intercepted a greater proportion of the photosynthetically active radiation, from an early stage of crop development and higher grain yield was produced in this orientation as compared to the north–south row orientation [47,146]. An east–west orientation of wheat and barley reduced the annual ryegrass seed production by 48% [47]. However, row orientation did not have a significant and consistent effect on crop yield and weed suppression in canola, field pea and lupin crops [146]. Hence, manipulating row orientation can also boost the competitive ability of crops in some situations against annual ryegrass populations.

### 5.4. Use of Allelopathic Crops

The phenomenon of allelopathy can be explored for weed management in cropping systems. Although it is hard to detach crop competition from allelopathic potential, studies have shown that certain crop cultivars suppress weeds due to their allelopathic properties. Wheat is a major grain crop in Australia and is known to be allelopathic against other crops and weeds [147]. Wu, et al. [54] reported that wheat straw reduced annual ryegrass germination by 4 to 73% and reduced root growth by 19 to 99%. Laboratory screening of wheat seedlings of 453 different accessions indicated that varying accessions inhibited the root growth of annual ryegrass from 10 to 91% [148]. Accessions were classified as strongly allelopathic where they inhibited root growth of annual ryegrass by >81% and weakly allelopathic where the inhibition was <45% [148]. Benzoxazinoids are key allelochemicals in wheat that suppress weeds and agricultural pathogens [149]. Several other phytotoxic secondary metabolites from wheat including *p*-hydroxybenzoic, vanillic, *cis-p*-coumaric, syringic, *cis*-ferulic, *p*-coumaric and *trans*-ferulic acids, were also reported to suppress annual ryegrass growth [150,151].

Annual ryegrass is also a key weed associated with canola in Australia and research has shown that canola and other related *Brassicaceae* species exhibit allelopathic properties [152]. Canola plants released potent allelochemicals in root growth medium, suppressing the root growth of annual ryegrass [152,153]. Advanced metabolomics analysis revealed that allelochemicals including sinapyl alcohol, *p*-hydroxybenzoic acid and pentahydroxyflavone were solely exuded from canola genotypes [153] that were superior in suppressing the root growth of annual ryegrass [152,154].

Control of annual ryegrass in the pasture phase is important to achieve the crop disease break, to reduce the competition of annual ryegrass in winter crops and to reduce the reliance on grass selective herbicides. Recently, alfalfa genotypes, at varying densities, were investigated for allelopathic impact on annual ryegrass in a laboratory bioassay [155]. These genotypes inhibited the elongation of annual ryegrass seedlings by 5 to 65%. Significant variation was observed in allelochemicals present in the root extracts and exudates across genotypes [155].

It is clear that there is considerable genetic variation between and within crops, particularly in canola and related Brassica species, for allelopathic activity against annual ryegrass. The allelopathic potential of certain crops and genotypes could be exploited for the ecological management of annual ryegrass in different cropping systems. It is also apparent that this variation presents an opportunity for breeding new cultivars with greater weed suppression ability.

### 5.5. Biological Control

Biological control has shown some potential in controlling herbicide-resistant biotypes of annual ryegrass. In a study from WA, Flores-Vargas and O’hara [156] reported that some strains of deleterious rhizosphere inhabiting bacteria reduced seedling growth and biomass production of annual ryegrass and wild radish (*Raphanus raphanistrum* L.), without any inhibitory effect on the yield of grapevine (*Vitis vinifera* L.). However, this has not been tested under broad scale cropping systems. *Pyrenophora semeniperda* is a potential bioherbicide in Australia [157]. It has been shown to reduce the emergence and seedling vigour of annual ryegrass, among other grass weeds in Australia. This biological control agent is practical for release in Australia, as it is already widely distributed in North and South America, South Africa, New Zealand and Egypt. However, the dry climate of Australia which does not always favour the growth of fungi pose a challenge [157].

Seed predation by ants could be an effective tool to reduce the surface seedbank of annual ryegrass, especially in no-till systems with high residue cover. In a study from WA, ants removed up to 100% of annual ryegrass seeds from the soil surface over the summer/autumn period [158]. *Pheidole hartmeyeri* Forel was reported as the most effective species of granivorous ants in removing the seeds of annual ryegrass [159]. Two other ant species (*Melophorus turneri* Forel. and *Monomorium rothsteini* Forel.) also have the potential to act as a biocontrol agent by removing annual ryegrass seeds. Seed predation was inconsistent over the field and further research is required to increase the spread of granivorous species. This may involve the inclusion of non-crop areas to encourage ant species or use of low disturbance tillage systems that leave ant nests intact during autumn crop seeding.

### 5.6. Harvest Weed Seed Control

Annual ryegrass retains the majority of its seeds until crop maturity. It has been estimated that 85 to 95% of seeds of annual ryegrass were retained at maturity, although 21 to 33% of heads were less than 10 cm high and unlikely to enter the harvester [40,44,160]. Innovative Australian growers and scientists have turned this attribute of annual ryegrass against itself by developing different grading systems that target annual ryegrass seeds so that they exit the harvester in the chaff fraction [161]. Different harvester systems have been developed and undergone consistent improvement over the past three decades. These systems differ in their operation but are similarly effective in reducing the annual ryegrass soil seedbanks (Table 5). With the ever-increasing problem of herbicide resistance in annual ryegrass, the adoption of HWSC systems has increased. According to a 2014 survey, 43% of Australian grain growers were using a form of HWSC. The highest adoption (30%) was recorded for a narrow windrow burning system, whereas only 1% of growers were using the Harrington seed destructor [162]. Importantly, 82% of growers indicated that they will consider using HWSC in the future [162].

Although HWSC has resulted in significant advances in the management of annual ryegrass, variable climatic conditions may decrease the seed retention by annual ryegrass, compromising its efficacy in some circumstances. Given the genetic diversity of annual ryegrass, there is the potential for this weed to adapt to this form of control. Therefore, it is important to integrate HWSC with cultural and chemical control options to maintain a diversity in the management programs that will delay the evolution of adaptive mechanisms in the weed populations.

### 5.7. Integrated Management

No single management tool can provide effective control of annual ryegrass on a sustainable basis. Therefore, integrating compatible and suitable control methods is important for managing prolific weeds like annual ryegrass [169]. Although work has occurred on the integration of management techniques, the number of studies reporting on true integrated weed management (IWM) programs against annual ryegrass is low. To avoid or delay resistance evolution to any and all forms of weed control in annual ryegrass, it is imperative to integrate weed control options, but particularly herbicides with non-chemical options [88,98,169,170]. The studies have shown that combining weed control methods that can affect weed growth and development in different ways do provide effective control (Table 6). Hence, the successful, long-term management of annual ryegrass depends on pragmatic IWM strategies. 

## 6. Lessons Learnt and Future Directions

Annual ryegrass has become the driver weed for weed management programs in most Australian cropping systems due to its widespread occurrence, high likelihood of herbicide resistance, and significant impact on crop yield and quality. Therefore, effective management of this species is the core of the overall success of any weed management program. Australian researchers and growers have made significant progress in understanding the resistance mechanisms and managing diverse populations of this problematic weed species. Continuous diversification (inclusion of multiple control methods in the system) and innovation (such as the development of HWSC systems) over the past few decades indicates that there have been some successes, but this weed remains a significant threat. Specifically, the following areas require further attention:

1. Annual ryegrass populations have shown significant flexibility in phenology by expanding their germination and growth ability over a wide range of environmental conditions, especially varying temperatures. This could have large implications in terms of further temporal and spatial spread of this weed across crop production systems in Australia. Therefore, a better understanding of underlying biological mechanisms for this potential adaptive evolution under changing management and climatic conditions is required.
2. Annual ryegrass populations have shown the ability to not only adapt to a wide range of herbicides but also to other management tactics. Therefore, it is important to continuously monitor its biology and ecology under a range of management selection pressures in order to discover potential adaptations at early stages. For example, research on the potential speed of annual ryegrass adaptation to HWSC and other forms of cultural control, is crucial to modify/improve the management programs accordingly.
3. Herbicide programs should be regularly assessed to make sure they are diversified to avoid or delay the evolution of resistance.
4. Management programs must be based on ‘diversity’ and a ‘system approach’ keeping in view the needs and limitations of individual farms and regions. There is a need for long-term seedbank and weed population dynamic studies that investigate the impacts of conservation cropping systems on different aspects of annual ryegrass biology.
5. Research on advancing/improving innovative weed control methods such as alternate non-chemical weed control methods is needed. Recent technological advances have enabled in-crop site-specific application of many alternative weed control techniques such as targeted herbicide application/optical spot spraying or mechanical weed removal. Further optimisation and adoption of such innovative tools should be prioritised.

## Figures and Tables

**Table 1:** Estimated burial depth of annual ryegrass seed, as a percent of total seed currently on the soil surface, by a range of tillage methods.

Purpose of Tillage	Tillage Method	Example	Percentage of Seed at Different Depths (cm)				
			0	0 to 1	1 to 5	5 to 10	10 to 20
Shallow soil cultivation, for burial of seed and crop residue	Rotary harrow	Phoenix Harrow; evenly distributes soil to 5 cm.	15	50	35	0	0
	Autumn tickle	Kelly chain; distributes 90% of surface seeds to a depth of 1 to 5 cm.	10	30	60	0	0
Full disturbance to address soil constraints	Multiple	Soil inversion using a mouldboard plough	1	1	1	1	96
Crop seeding	Minimum tillage	Direct drill with full cut cultivation; tynes cultivate the soil to 5 cm and provide a disturbed seed bed.	10	50	39	1	0
	Zero tillage	Disc seeding: flat discs are used to create an opening in the soils (to 5 cm) which is followed by a tyne to deliver seed and fertiliser into the slot, often followed by a press wheel to close the slot.	95	2	3	0	0
	No-tillage	Knifepoint seeding; narrow tynes commonly referred to as knife points resulting in 5 to 20% cultivation of the soil surface to 5 cm. Knife points will throw a small amount of soil across the surface, effectively burying the surface seed.	10	80	10	0	0

Source: Weed Seed Wizard decision support tool [16].

**Table 4:** Impact of various physical and cultural methods on annual ryegrass control.

Cultural Method	Brief Description	State	Control Achieved *	References
Grazing	Grazing the field at an optimum stocking rate, so sheep can readily graze all the plant parts, especially seed heads of annual ryegrass.	Victoria (Vic)	80% reduction in density	[100]
		WA	95% reduction in seedset	[101]
		WA	90% reduction in density	[102]
		WA	93% reduction in seedset	[103]
Hay	Cutting the excess pasture for hay consumption to reduce the density and seed production of annual ryegrass.	Vic	84% reduction in density	[100]
		NSW	45% reduction in density	[90]
		WA	95% reduction in density	[104]
Silage	Cutting the pasture for silage production in order to reduce annual ryegrass density.	NSW	91% reduction in density	[90]
		WA	98% reduction in density	[104]
Burning	Burning pastures or stubbles can destroy the annual ryegrass infestation completely and kill the weed seeds present on the soil surface.	Vic	66% reduction in density	[100]
		Vic	35 to 57% reduction in density	[105]
		SA	60% reduction in seed number	[106]
		NSW	Up to 98% reduction in seedset	[107]
		WA	82% reduction in seed number	[108]
Inversion/Mouldboard ploughing	Deep inversion tillage to put the weed seeds to a greater depth from where they cannot germinate/emerge.	Vic	73% reduction in density	[100]
		WA	>95% reduction in density	[109]
		WA	96% reduction in density	[110]
		WA	100% reduction in seedbank after four years of burial at 15 cm depth	[111]
Autumn tickle/Shallow cultivation	Shallow tillage stimulates germination of weed seeds by putting them in a physical depth of 1 to 3 cm, and then controlling them effectively.	Vic	59% reduction in density	[100]
		WA	51 to 63% reduction in density	[112]
		NSW	37% reduction in density	[113]
Delayed sowing	Crop plating is delayed by up to four weeks beyond the optimum time to maximize emergence of annual ryegrass, and then controlling them through direct cultivation or use of burndown herbicides.	SA	52% reduction in weed density with three weeks delay in sowing	[106]
		SA	11 to 30% reduction in weed density with each week delay in sowing	[88]
Green manuring	Green manuring is the incorporation of green crop residues into the soil with a mechanical implement like disc plough.	NSW	97% reduction in density	[90]
		WA	94% reduction in density	[114]
		WA	80% reduction in density	[115]
		WA	98% reduction in density	[104]
Brown manuring	Brown manuring is the desiccation of weeds and crops at the flowering stage by using burndown herbicides to reduce the seed production of target weeds.	WA	79% reduction in density	[114]
		WA	98% reduction in density	[104]
Mulching	Mulching involves slashing or mowing the pasture or crops and then laying it on the soil surface to ensure more soil contact and reduce moisture loss through evaporation.	WA	82% reduction in density	[114]
Mowing	Physical cutting of annual ryegrass plants with a mechanical implement before the seed production stage.	WA	98% reduction in density	[104]
Swathing	The harvesting of a crop at maturity through physical (windrowing) or chemical means (desiccation) is called swathing.	WA	45% reduction in density	[104]

* In comparison with untreated control treatment.

**Table 5:** Different HWSC systems and their potential for annual ryegrass seed kill.

HWSC System	Brief Description	State	Seed-Kill (%)	References
Chaff carts	During harvesting, chaff carts can be towed just behind the header to gather the chaff material, which is either burnt in the coming autumn or utilized as livestock feed.	SA	52	[106]
		SA	60 to 80	[3]
		WA	75 to 85	[77]
		WA	32 to 75	[163]
		WA, Vic, NSW	60	[164]
Narrow windrow burning	A chute can mount to the rear of the harvester which concentrates the entire chaff material into the narrow windrow (50 or 60 cm in depth) for onward stubble burning in the favourable autumn season.	WA	99	[165]
		WA	30 to 90	[163]
		WA, Vic, NSW	60	[161]
Baler direct system	This HWSC system comprises of a big square baler which can attach to the grain harvester in collecting chaff material and then transforming it into bales.	WA	95	[77]
Chaff-tramlining and chaff-lining	In this system, the chaff material is concentrated into narrow rows of 20 to 30 cm width. If this material is confined in narrow-rows on the specific wheel tracks, it is known as chaff-tramlining and if it is confined amid stubble rows, it is called chaff-lining. These chaff lines are constructed by equipment attached to the rear of the harvester and used for collection and storage of chaff material.	Queensland (Qld), NSW	73 to 89	[166]
		Qld, NSW	59	[166]
Harrington seed destructor	This system comprises of cage mill which processes the chaff material for the destruction of weed seeds and then discard them directly into chaff delivery systems.	WA	95	[163]
		WA	35 to 90	[163]
		WA, Vic, NSW	60	[162]
Integrated Harrington seed destructor	The is a new version of the Harrington seed destructor which is based on the unique design of impact mill which can mount on the back of the harvester to process the chaff material and kill the weed seeds.	-	93	[167]
		WA	88 to 98	[168]

**Table 6:** Examples of effective integrated management of annual ryegrass.

Integrated Management	State	Control Achieved	References
Grazing + knockdown herbicide	WA	80% reduction in seedbank	[102]
Grazing + knockdown herbicide	Vic	85% reduction in density	[105]
Crop topping + pre-emergent herbicide	SA	99% reduction in seedset	[171]
Autumn tickle + delayed sowing for six weeks	WA	73% reduction in density	[112]
Silage + paraquat	WA	95% reduction in seedset	[172]
Narrow row spacing + harvest weed seed control	WA	100% reduction in seedset	[52]

## Data Availability

Not applicable.
